# Increased daytime and awakening salivary free aldosterone in essential hypertensive men

**DOI:** 10.3389/fcvm.2024.1335329

**Published:** 2024-06-25

**Authors:** Angelina Gideon, Roland von Känel, Cathy Degroote, Livia Thomas, Claudia Zuccarella-Hackl, Roland Wiest, Petra H. Wirtz

**Affiliations:** ^1^ Biological Work and Health Psychology, University of Konstanz, Konstanz, Germany; ^2^ Department of Consultation-Liaison Psychiatry and Psychosomatic Medicine, University Hospital Zurich, University of Zurich, Zurich, Switzerland; ^3^ Support Center of Advanced Neuroimaging, Institute of Diagnostic and Interventional Neuroradiology, University Hospital Bern, University of Bern, Bern, Switzerland; ^4^ Centre for the Advanced Study of Collective Behaviour, University of Konstanz, Konstanz, Germany

**Keywords:** salivary free aldosterone, awakening response, daytime levels, essential hypertension, blood pressure

## Abstract

**Background:**

While aldosterone plays an important role in blood pressure regulation, its role in essential hypertension (EHT) remains unclear. Here, we systematically investigated the secretion of biologically-active free aldosterone in saliva in response to awakening (AldAR) and during the day (AldDay) in EHT compared to normotensive controls (NT).

**Methods:**

In 30 men with EHT and 30 age-matched NT, AldAR saliva samples were collected immediately after awakening and 15, 30, 45, and 60 min thereafter and AldDay samples were collected from 08:30–22:00 h on two consecutive days.

**Results:**

Over the course of the day, men with EHT had higher repeated AldDay levels compared to NT (*p *=* *.002) with higher concentrations in the morning hours (*p*'s ≤ .047), a steeper decline over the course of the day (*p*'s ≤ .018), and similar concentrations in the evening (*p*'s ≥ .21). Regarding AldAR, we observed higher concentrations in EHT at awakening (*p *=* *.017) and borderline higher concentrations at 15 min (*p *=* *.086). No differences were found 30–60 min after awakening (*p*'s ≥ .34). Analyses with repeated and aggregated AldAR levels resulted in borderline significantly higher free aldosterone in EHT (*p*'s ≤ .077). Complementary analyses confirmed linear associations between higher blood pressure and higher AldAR and AldDay levels.

**Conclusions:**

Our data point to elevated salivary free aldosterone secretion in EHT over the course of the day, particularly in the morning hours. As the free aldosterone fraction is considered biologically active, our data may point to a biological mechanism underlying EHT.

## 1 Introduction

Arterial hypertension (HT) is characterized by persistent elevation in arterial blood pressure (BP) affecting more than 30% of adults worldwide (1) and is one of the major risk factors for cardiovascular disease (CVD) (2, 3). The majority of HT patients is diagnosed with essential hypertension (EHT) with the cause for their condition being multifactorial (4).

One of the factors involved in BP regulation is the mineralocorticoid hormone aldosterone. Aldosterone is released from the zona glomerulosa of the adrenal gland and is mainly regulated by the renin–angiotensin–aldosterone–system (RAAS), by the adrenocorticotropic hormone (ACTH), and by potassium concentrations (5). Aldosterone regulates extracellular space volume and thus BP by modulating the sodium-potassium balance (5, 6). Excess of aldosterone and activation of the receptor that mediates aldosterone effects, i.e., the mineralocorticoid receptor, has been associated with the development and progression of CVD, in particular of coronary heart disease and its underlying process atherosclerosis, but also with CVD risk factors, in particular with HT (5, 7). Moreover, prospective studies showed that with higher plasma or serum aldosterone levels, the risk of a rise in BP and of developing HT increased after four (8, 9) and five years (10).

Based on this, high aldosterone levels may be present in at least one subgroup of previously cataloged EHT patients. So far, most studies assessed basal aldosterone levels in EHT either by single time measurements from plasma and serum samples or by measuring aggregated urinary aldosterone excretion over 24 h. Interestingly, these studies report inconsistent results: while some studies found higher basal plasma and serum aldosterone levels in (untreated) EHT as compared to normotensive (NT) controls (11, 12, 13), others report comparable basal plasma, serum, and urinary aldosterone levels in (untreated) EHT patients and NT (11, 14–17). Similarly, in NT and/or HT participants, higher plasma aldosterone levels have been associated with higher BP levels (as a continuous variable) in some studies (12, 18, 19) while in other studies aldosterone did not relate to BP (20, 21). Notably, in addition to a stimulation-dependent, i.e., stress-induced, high variability (14, 22, 23), basal aldosterone follows a circadian rhythm with highest aldosterone levels in response to awakening, i.e., in the morning, that decrease during the day reaching lowest levels in the evening (24–28). Thus, circadian aldosterone secretion can only incompletely be captured by a single measurement, especially given the aldosterone awakening response (AldAR) within the first hour after awakening (26). So far, four studies compared circadian plasma aldosterone rhythms between HT patients and NT controls. They repeatedly measured daytime aldosterone every two (29), four (30, 31), and six hours (32) but did not report significant differences between HT and controls. Notably, one of these studies reported elevated aldosterone plasma levels in subgroups of EHT participants (32).

Aldosterone measurement from blood allows for the assessment of total aldosterone, including both the bound and unbound fractions of aldosterone. While the majority of aldosterone in an organism is bound predominantly to corticosteroid-binding-globulin (CBG) and albumin, about 35%–40% of total aldosterone is unbound and thus capable of exerting the biological effects of aldosterone (33, 34). Recently, it was shown that aldosterone can reliably be measured in saliva (35) reflecting about 80% of the biologically active free aldosterone fraction in plasma (28). Free aldosterone levels in saliva correlate strongly with total aldosterone plasma levels (22) and are independent of saliva flow (35). Benefits of saliva sampling include the non-invasive collection and that samples can be stored at ambient temperatures allowing for self-administered repeated sampling in natural environment. Notably, urine comprises only about 0.5% of the daily secreted free aldosterone (36) while the metabolites aldosterone 18-glucuronide and tetrahydroaldosterone represent the majority of excreted aldosterone (37).

To date, one study assessed diurnal salivary aldosterone in EHT and NT controls (38). In that study, aldosterone was assessed twice at variable, i.e., non-standardized timepoints—once between morning and noon (between 08:00–12:00 h) and once in the evening (20:00–24:00 h). EHT patients were found to have higher salivary aldosterone levels than NT controls. Independent from time of sampling and based on the two assessment timepoints EHT participants did not show a decline during the day (38). Admittedly, the varying sampling times might have a considerable impact on the results as aldosterone levels were shown to exhibit high variability in the morning hours and particularly after awakening (26). To the best of our knowledge, there is no study investigating circadian salivary aldosterone levels with standardized and more frequent sampling times in EHT and NT.

We therefore set out to systematically investigate salivary aldosterone daytime levels (AldDay) and AldAR in EHT and NT. Saliva samples were collected at standardized timepoints from awakening until bedtime in participants’ natural environment in accordance with the guidelines for salivary cortisol assessment (39). Further, we controlled for potential confounding variables including sleep-related variables as well as age and BMI. We hypothesized that participants with EHT will show overall higher salivary aldosterone levels as compared to NT.

## 2 Methods

### 2.1 Study participants and assessment of hypertension

The current project is part of a series of studies assessing psychoneurobiological mechanisms in EHT (40–42). The study program was approved by the ethics committee of the State of Bern, Switzerland and all participants provided written informed consent.

As previously described in more detail (40–42), we recruited apparently healthy, nonsmoking, medication-free HT and NT men by aid of the Swiss Red Cross of the State of Bern. Exclusion criteria, as verified in a telephone interview, using an extensive health questionnaire, were: regular strenuous exercise, alcohol and illicit drug abuse, atopic diathesis and allergies, chronic obstructive pulmonary disease, liver and renal diseases, rheumatic diseases, HIV, cancer, psychiatric and neurological diseases, and current infectious disease.

For classification of HT and NT, eligible participants were instructed to record their BP levels in a seated position after a 15-min rest twice a day on three days at home by sphygmomanometry (Omron M6; Omron Healthcare Europe B.V., Hoofdorp, Netherlands). From the available measurements the average home BP was computed for each participant. According to recommendations for home BP measurements (43), HT was defined as average systolic BP ≥ 135 mmHg and/or average diastolic BP ≥ 85 mmHg and NT was defined as average home systolic BP < 135 mmHg and diastolic BP < 85 mmHg. This preliminary classification was verified by trained personnel at the laboratory appointment with three additional BP measurements. Participants with an average systolic BP of ≥ 140 mmHg and/or an average diastolic BP of ≥ 90 mmHg were classified as HT (43). In addition, to screen for potential secondary HT, we measured serum creatinine, calcium, sodium, and potassium levels in eligible HT participants, and normal ranges were verified by a board-certified internist. None of our participants provided indication of a diagnosis of primary aldosteronism (44, 45). Exclusion criteria for HT/NT classification comprised incongruent home and laboratory BP measurements, or evidence for secondary HT. Home BP measurements of two HT participants were missing who however indicated in the health questionnaire that they had a previous diagnosis of EHT. Moreover, sodium, potassium, and calcium could not be analysed in four HT participants.

Based on our previous studies assessing salivary endocrine measures in HT (14, 42, 46), we expected group differences in salivary aldosterone levels between EHT and NT of small to medium effect size. A-priori power calculation revealed that the total sample size to detect a conservatively expected effect size of *f *=* *.15 with a power of .80, an *α* = .05, and a correlation among repeated measures of *r *=* *.50, is *N *=* *56 for the total sample. For the purpose of salivary aldosterone assessment, participants were considered eligible if enough saliva was available for complete aldosterone AldAR and AldDay profiles (≤ 2 missings per profile), if participants adhered to the sampling protocol (with an interval of less than 10 min between awakening and the first saliva sample, and not more than 75 min between the first and the 60 min saliva sample (39), and if participants provided demographic, physiological, and sleep-related variables. Based on our a-priori power calculation and due to limited funding, we restricted the sample size of eligible EHT participants to *n *=* *30 and selected 30 age-matched (≤ 2 years) healthy NT participants whose saliva samples were then analyzed.

### 2.2 Design and procedure

The study comprised a laboratory appointment at the University Hospital of Bern and two days with saliva assessment at participants' homes. In anticipation of the laboratory appointment, participants were instructed to abstain from caffeine and alcohol consumption for 24 h and to consume a semi-standardized breakfast. Upon arrival at the laboratory at 08:00 h, participants completed questionnaires, their body height and weight were measured, and blood samples for secondary HT screening were collected. BP was measured three times under resting conditions after at least 15 min sitting before each measurement by means of sphygmomanometry (Omron M6; Omron Healthcare Europe B.V., Hoofdorp, Netherlands). Further, participants received study materials and written instructions for the saliva assessment day.

Participants were instructed to collect saliva at home on two consecutive work days using salivette devices (Sarstedt, Rommelsdorf, Germany). The instructions further included refraining from strenuous physical activity while normal daily routines were allowed. Self-reports of wake-up times, sleep duration, and saliva collection times were complemented by electronic monitoring devices (Medication-Event-Monitoring-System, MEMS-TrackCap, Aardex Group, Switzerland). Moreover, participants were requested to wake up at the latest at 08:00 h, to stay in bed for the first 15 min, to abstain from breakfast during the first 30 min, i.e., until after collection of the salivette 30 min after awakening. Participants were asked to refrain from drinking coffee or juicy beverages in the subsequent breakfast. Also, participants were instructed to not brush their teeth during the first hour after awakening and to clean their mouth with water prior to each saliva collection.

To assess the AldAR, the first saliva sample was collected immediately after awakening and 15, 30, 45, and 60 min after awakening. AldDay samples were taken at 08:30, 11:00, 16:00, 20:00, and 22:00 h. The collected saliva samples were stored in participants’ refrigerators until the return to the laboratory where they were stored at −20°C until analyses.

### 2.3 Aldosterone analyses

At the University of Konstanz, we analyzed AldAR and AldDay of the selected EHT and NT participants from frozen saliva samples that were collected from 11/2011 to 10/2015. For aldosterone analyses, samples were thawed and centrifuged at 2,500 g for 10 min (Heraeus Megafuge 40R, Thermo Fisher Scientific, Langenselbold, Germany). Aldosterone concentrations were measured in duplicates using a commercially available enzyme-linked immunosorbent assay (ELISA, “Aldosterone ELISA”, RE52301, IBL International GmbH, Hamburg, Germany); detection limit was 12.07 pg/ml and mean inter- and intra-assay coefficients of variance were 4.3% and 5.3%, respectively. Levels below the detection limit (i.e., five samples) were replaced by half the detection limit (47).

### 2.4 Statistical analyses

Statistical analyses were performed using SPSS (Version 28.0) statistical software packages for MacIntosh (IBM-SPSS Statistics, Chicago IL, USA). All tests were two-tailed with level of significance at *p *<* *.05. Data are presented as mean ± standard error of the mean (*mean* ± *SEM*).

For aldosterone levels and data relating to aldosterone sampling (i.e., awakening times, sleep duration, and sampling times) we calculated mean levels of the two sampling days. In 20 participants, aldosterone data of only one day was available and we used the sampling data of that day.

We computed aggregated parameters that comprise diurnal slopes using rise over run (48) from awakening to 22:00 h (slope_awake_), and from 08:30–22:00 h (slope_max_) as well as areas under the curve (49) with respect to ground (AUCg) for AldDay to capture the total secretion and with respect to increase (AUCi) for AldAR to capture the secretion starting from the respective awakening levels. Mean arterial blood pressure (MAP) was calculated by the formula (2/3 * mean diastolic BP) + (1/3 * mean systolic BP) and BMI was calculated by the formula kg/m^2^.

All data were tested for normal distribution and homogeneity of variance using Kolmogorov–Smirnov and Levene's tests. In order to approximate a normal distribution, the variables age, time of awakening, mean home systolic BP, mean home MAP, and aldosterone levels were ln-transformed. For reasons of clarity, we depict untransformed data in figures and tables.

We compared groups in terms of demographic and biological measures using univariate analyses of variance (ANOVA). To test whether EHT participants differed from NT controls in terms of AldAR and AldDay, we calculated univariate AN(C)OVAs with group as the independent variable and aldosterone levels immediately after awakening as the dependent variable. Then, we performed repeated measures AN(C)OVAs with group as the independent variable and the respective AldAR and AldDay levels as repeated dependent variables. Analyses were performed without and with controlling for age, BMI, awakening time, and sleep duration as potentially confounding covariates (26). Complementary testing comprised univariate AN(C)OVAs with AUC measures as dependent variables. Post-hoc testing comprised univariate AN(C)OVAs with group as the independent variable, and with separate aldosterone measurements and diurnal slopes as dependent variables, again without and with the above-mentioned covariates. Moreover, repeated measures ANOVAs were performed in each subject group separately where relevant. Further, we tested for linear associations by repeating the above analyses using MAP as continuous variable. We applied Huynh-Feldt correction for repeated measures. Effect sizes are reported where appropriate as partial eta squared (*η_p_*^2^) and Cohen's *f*. Effect size conventions are: small: *η_p_*^2^ ≥ .01, *f *=* *.10; medium: *η_p_*^2^ > .06, *f *=* *.25; large: *η_p_*^2^ > .14, *f *=* *.40 (50).

## 3 Results

### 3.1 Participants' characteristics

Participants' characteristics are depicted in Table 1. Mean age was 52.87 ± 1.15 years and mean BMI was 26.87 ± 0.44 kg/m^2^. Self-reported awakening times and sleep duration were on average 06:00 h and 7 h, 6 min, respectively. While the groups did not differ in age, awakening-time, or sleep duration (*p*'s ≥ .15), EHT had significantly higher BMI and blood pressure levels (*p*'s ≤ .003).

**Table 1:** Characteristics of the 60 participants studied.

	Essential hypertensives (*n* = 30) *mean* ± *SEM* (range)	Normotensives (*n* = 30) * mean* ± *SEM* (range)	*p*	*η_p_*^2^/*f*
Age (years)	52.87 ± 1.66 (37–69)	52.87 ± 1.62 (38–68)	.99	
BMI (kg/m^2^)	28.14 ± 0.67 (20.35–35.25)	25.60 ± 0.50 (19.78–30.85)	**.003**	.14/.40
Home MAP (mmHg)	107.66 ± 1.24 (99.01–121.50), *n* = 28	88.57 ± 0.81 (80.56–95.94)	**<.001**	.75/1.74
Mean home sBP (mmHg)	145.76 ± 1.87 (125.67–162.33), *n* = 28	122.19 ± 0.97 (110.00–133.83)	**<.001**	.71/1.56
Mean home dBP (mmHg)	88.61 ± 1.31 (78.50–103.00), *n* = 28	71.76 ± 0.93 (60.83–80.00)	**<.001**	.67/1.42
Office MAP (mmHg)	116.01 ± 1.71 (103.11–139.89)	93.91 ± 1.42 (75.33–104.56)	**<.001**	.63/1.31
Mean office sBP (mmHg)	154.51 ± 2.41 (129.33–189.67)	125.99 ± 1.61 (109.33–139.67)	**<.001**	.62/1.29
Mean office dBP (mmHg)	96.76 ± 1.51 (80.00–115.00)	77.87 ± 1.45 (58.33–89.00)	**<.001**	.59/1.19
Creatinine (µmol/L)	83.33 ± 1.91 (66.00–103.00)	** **		** **
Sodium (mmol/L)	141.08 ± 0.34 (138.00–145.00), *n* = 26	** **		** **
Calcium (mmol/L)	2.38 ± 0.02 (2.27–2.48), *n* = 26	** **		** **
Potassium (mmol/L)	4.25 ± 0.07 (3.80–4.70), *n* = 26	** **		** **
Awakening time (hh:mm)	06:01 ± 8 min (04:20–07:27)	05:59 ± 7 min (04:40–06:55)	.87	** **
Sleep duration (h, min)	7 h, 13 min ± 7 min (6 h, 15 min–8 h, 30 min)	6 h, 57 min ± 9 min (4 h, 56 min–8 h, 1 min)	.15	** **

*SEM*, standard error of the mean; BMI, body mass index; MAP, mean arterial blood pressure; sBP, systolic blood pressure; dBP, diastolic blood pressure.

Significant values (*p* < .05) are highlighted in bold.

### 3.2 AldDay

#### 3.2.1 Group differences between EHT and NT

**Figure 1:**
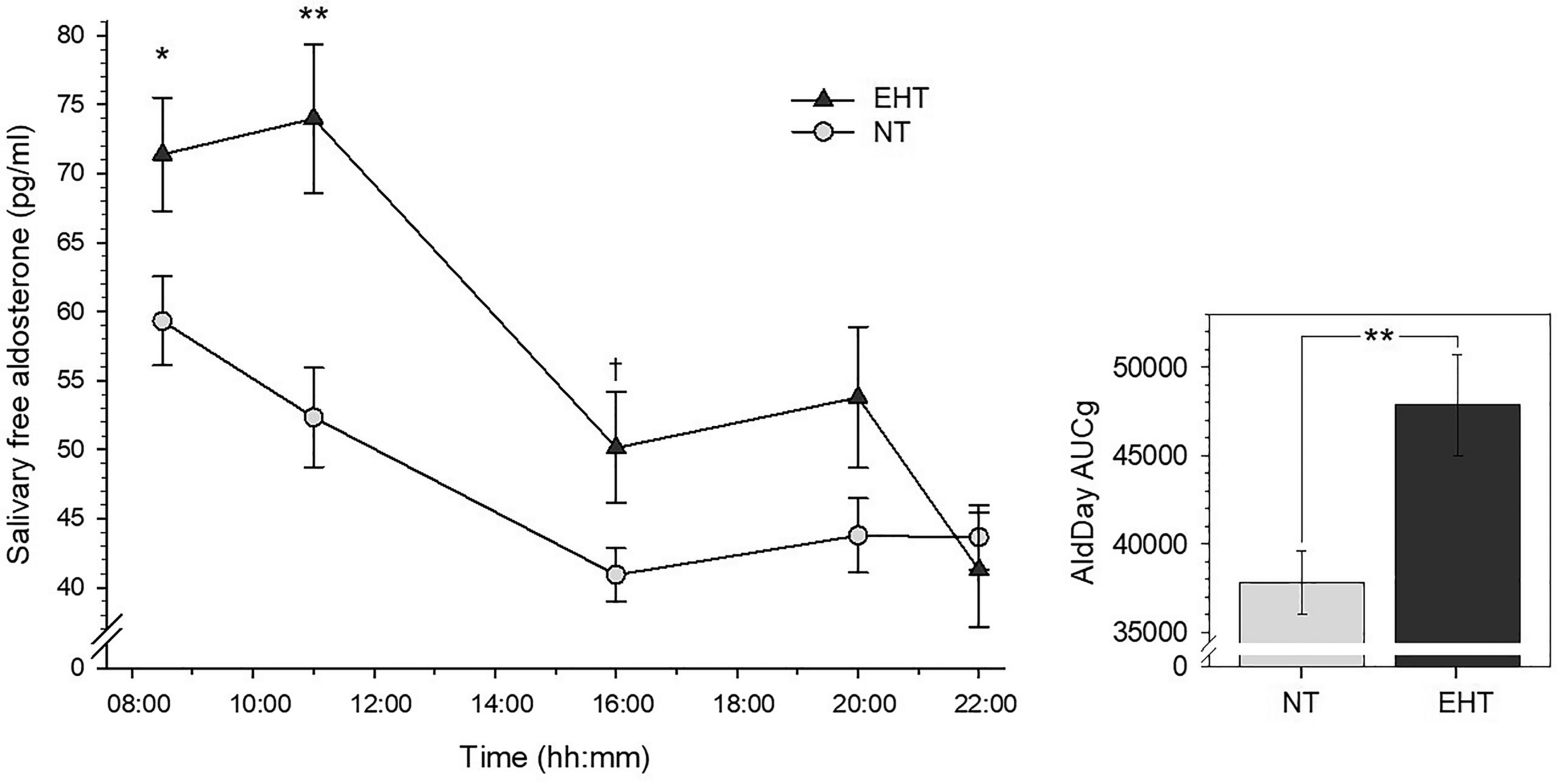
Left-side plot depicts salivary free aldosterone daytime levels in essential hypertensive (EHT) and normotensive (NT) participants (*mean* ± *SEM*). Right-side plot depicts aldosterone daytime AUCg's (*mean* ± *SEM*). **p < .01; *p < .05; †*p* < .10.

Repeated measures AN(C)OVAs with AldDay levels (08:30–22:00) revealed significant interactions between AldDay and group [*F* (3.13, 181.70) = 4.88, *p *=* *.002, *η_p_*^2 ^=^ ^.08, *f *=* *.29; with confounders controlled: *F* (3.36, 181.63) = 4.70, *p *=* *.002, *η_p_*^2 ^=^ ^.08, *f *=* *.29] with overall borderline significantly higher aldosterone levels in EHT [main effect group: *F* (1, 58) = 3.04, *p *=* *.087, *η_p_*^2 ^=^ ^.05, *f *=* *.23; with confounders: *p *=* *.27; see Figure 1]. Complementary univariate AN(C)OVAs with aggregated aldosterone levels similarly revealed significantly higher AldDay AUCg's (Figure 1) in the EHT group as compared to the NT group [*F* (1, 58) = 8.91, *p *=* *.004, *η_p_*^2^ = .13, *f *=* *.39; with confounders: *F* (21, 54) = 6.31, *p *=* *.015, *η_p_*^2 ^=^ ^.11, *f *=* *.35].

Post-hoc univariate AN(C)OVAs (see Table 2 for detailed statistics) with individual aldosterone concentrations showed significantly higher levels in the EHT group at 08:30 h (*p* = .047, *η_p_*^2 ^=^ ^.07) although not independent of confounders (*p* = .12) and at 11:00 h (*p* = .002, *η_p_*^2 ^=^ ^.16; with confounders: *p* = .011, *η_p_*^2 ^=^ ^.11) as compared to NT. At 16:00 h, aldosterone concentrations were borderline significantly higher in EHT (*p* = .089, *η_p_*^2 ^=^ ^.05) but not independent of confounders (*p* = .13). At 20:00 and 22:00 h, there were no significant group differences without control for confounders (*p*'s ≥ .21) and borderline significantly higher levels in NT at 22:00 h when controlled for confounders (*p* = .092, *η_p_*^2 ^=^ ^.05). Slopes_awake_ as well as slopes_max_ were significantly steeper in the EHT participants as compared to NT (slopes_awake_: *p *=* *.018, *η_p_*^2^ = .09; controlled for confounders: *p *=* *.017, *η_p_*^2 ^=^ ^.10; slopes_max_: *p *=* *.015, *η_p_*^2 ^=^ ^.10; controlled for confounders: *p *=* *.011, *η_p_*^2 ^=^ ^.11).

**Table 2:** Post-hoc test results in terms of group-based (EHT vs. NT) and linear (associations with MAP) analyses for **AldDay** level*s.*

	AldDay Parameters	[*df_Num_, df_Den_*]	*F*	*p*	*η* ^2^ *_p_*	*f*
EHT vs. NT	08:30	[1, 58]	4.11	**.047** (.12)	.07	.27
	11:00	[1, 58] (1, 54)	11.11 (6.92)	**.002** **(.011)**	.16 (.11)	.44 (.36)
	16:00	[1, 58]	2.99	.089 (.13)	.05	.23
	20:00			.29 (.62)		
	22:00			.21 (.092)		
	Slopes_awake_	[1, 58] (1, 54)	5.98 (6.01)	**.018** **(.017)**	.09 (.10)	.32 (.33)
	Slopes_max_	[1, 58] (1, 54)	6.22 (6.89)	**.015** **(.011)**	.10 (.11)	.33 (.36)
Linear associations with MAP	08:30	[1, 58] (1, 54)	4.90 (3.19)	**.031** (.080)	.08 (.06)	.29 (.24)
	11:00	[1, 58] (1, 54)	10.43 (7.12)	**.002** **(.010)**	.15 (.12)	.42 (.36)
	16:00	[1, 58] (1, 54)	4.73 (3.26)	**.034** **(.044)**	.08 (.07)	.28 (.28)
	20:00			.12 (.26)		
	22:00			.37 (.19)		
	Slopes_awake_	[1, 58] (1, 54)	5.46 (5.90)	**.023** **(.019)**	.09 (.10)	.31 (.33)
	Slopes_max_	[1, 58] (1, 54)	7.99 (8.92)	**.006** **(.004)**	.12 (.14)	.37 (.41)

Statistical values are presented without (and with) the covariates age, BMI, awakening time, and sleep duration; AldDay, salivary aldosterone daytime levels (08:30–22:00); EHT, essential hypertension; MAP, mean arterial pressure; NT, normotension; df_Num_, degrees of freedom numerator; df_Den_, degrees of freedom denominator.

Significant values (*p* < .05) are highlighted in bold.

#### 3.2.2 Linear associations with MAP

To test for linear associations, we complemented our group-based analyses and repeated the above described analyses using MAP as continuous variable instead of group. MAP was significantly associated with higher repeated AldDay levels (interaction MAP-by-time: *F* (3.09, 179.07) = 4.03, *p *=* *.008, *η_p_*^2 ^=^ ^.07, *f *=* *.26; with confounders: *F* (3.29, 177.72) = 4.07, *p *=* *.006, *η_p_*^2 ^=^ ^.07, *f *=* *.27). Correspondingly, higher MAP was significantly associated with higher AUCg's [*F* (1, 58) = 12.08, *p *<* *.001, *η_p_*^2 ^=^ ^.17, *f *=* *.46; with confounders: *F* (1, 54) = 9.49, *p *=* *.003, *η_p_*^2 ^=^ ^.15, *f *=* *.42].

Post-hoc testing (Table 2) showed higher MAP to significantly relate to higher aldosterone levels at 08:30 (*p *=* *.031, *η_p_*^2 ^=^ ^.08; with confounders: *p *=* *.080, *η_p_*^2 ^=^ ^.06), at 11:00 (*p *=* *.002, *η_p_*^2 ^=^ ^.15; with confounders: *p *=* *.010, *η_p_*^2 ^=^ ^.12), and at 16:00 h (*p *=* *.034, *η_p_*^2 ^=^ ^.08; with control for confounders: *p *=* *.044, *η_p_*^2 ^=^ ^.07). There were no associations between MAP and aldosterone levels at 20:00 and 22:00 h (*p*'s ≥ .12). Higher MAP significantly related to steeper diurnal slopes (slopes_awake_: *p *=* *.023, *η_p_*^2 ^=^ ^.09; with control: *p *=* *.019, *η_p_*^2 ^=^ ^.10; slopes_max_: *p *=* *.006, *η_p_*^2 ^=^ ^.12; with control: *p *=* *.004, *η_p_*^2 ^=^ ^.14).

### 3.3 Awakening aldosterone

#### 3.3.1 Group differences between EHT and NT

**Figure 2:**
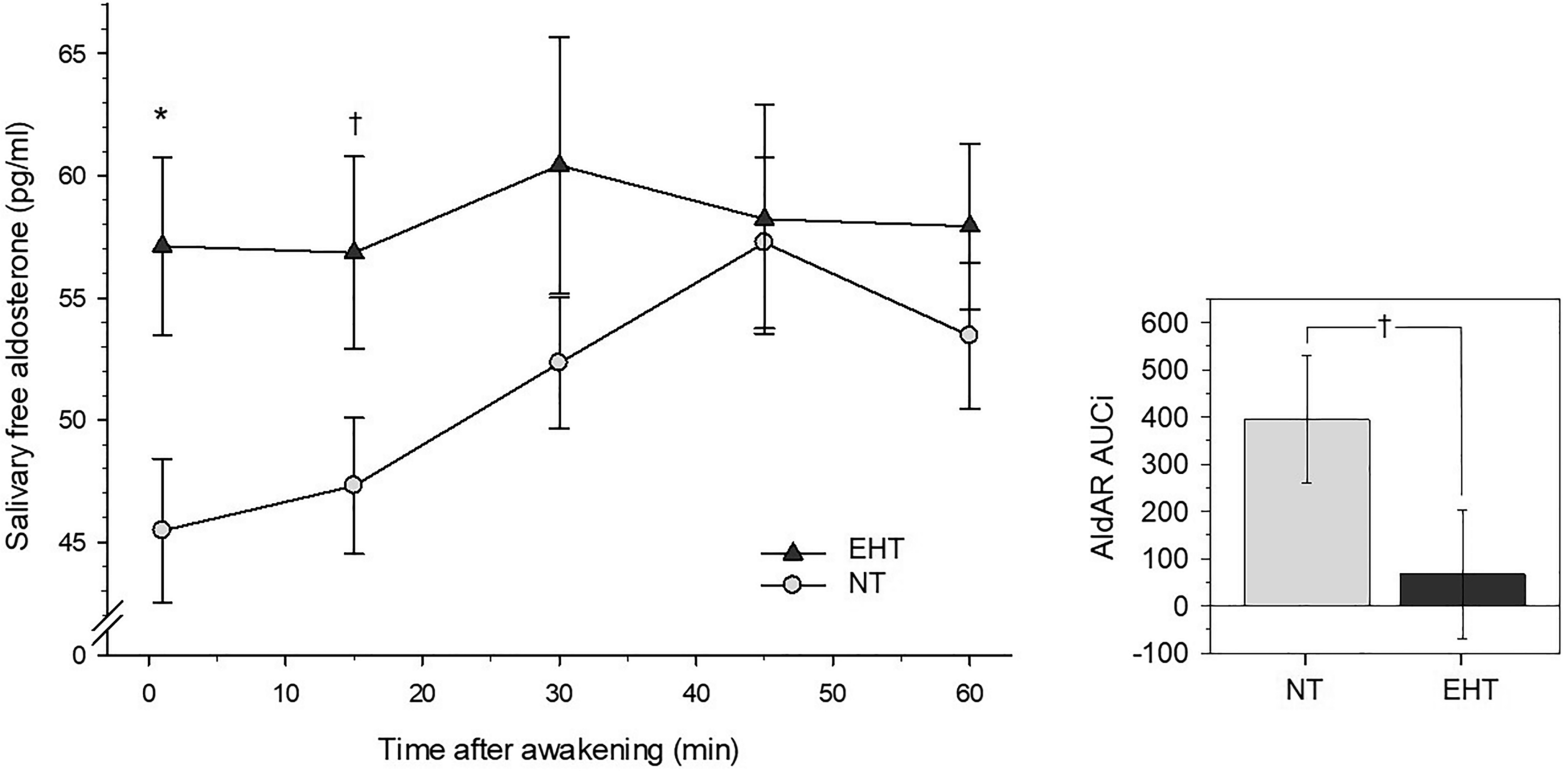
Left-side plot depicts salivary free aldosterone awakening response in essential hypertensive (EHT) and normotensive (NT) participants (*mean* ± *SEM*). Right-side plot depicts aldosterone awakening AUCi's depicting changes from awakening levels (*mean* ± *SEM*). *p < .05; †*p* < .10.

Immediately after awakening, EHT showed significantly higher aldosterone levels [*F* (1, 58) = 6.08, *p *=* *.017, *η_p_*^2 ^= .10, *f *=* *.32; controlling for confounders: *F* (1, 54) = 4.66, *p *=* *.035, *η_p_*^2 ^=^ ^.08, *f *=* *.29] as compared to NT (see Table 3 and Figure 2).

**Table 3:** Baseline and post-hoc test results in terms of group-based (EHT vs. NT) and linear (associations with MAP) analyses for **AldAR** level*s.*

	AldAR parameters	[df_Num_, df_Den_]	*F*	*p*	η^2^*_p_*	*f*
EHT vs. NT	0 min	[1, 58] (1, 54)	6.08 (4.66)	**.017** **(.035)**	.10 (.08)	.32 (.29)
	15 min	[1, 58]	3.04	.086 (.20)	.050	.23
	30 min			.34 (.57)		
	45 min			.88 (.96)		
	60 min			.40 (.35)		
Linear associations with MAP	0 min	[1, 58] (1, 54)	4.55 (3.64)	**.037** (.062)	.07 (.06)	.28 (.26)
	15 min			.30 (.14)		
	30 min			.25 (.17)		
	45 min			.16 (.23)		
	60 min			.28 (.29)		

Statistical values are presented without (and with) the covariates age, BMI, awakening time, and sleep duration; AldAR, salivary aldosterone awakening response (0–60 min); EHT, essential hypertension; MAP, mean arterial pressure; NT, normotension; df_Num_, degrees of freedom numerator; df_Den_, degrees of freedom denominator.

Significant values (*p* < .05) are highlighted in bold.

#### 3.3.2 Linear associations with MAP

Higher MAP was associated with higher aldosterone levels at awakening [*F* (1, 58) = 4.55, *p *=* *.037, *η_p_*^2 ^=^ ^.07, *f *=* *.28]. The association was attenuated to borderline significance after controlling for confounders [*F* (1, 54) = 3.64, *p *=* *.062, *η_p_*^2 ^=^ ^.06, *f *=* *.26] (see Table 3).

### 3.4 AldAR

#### 3.4.1 Group differences between HT and NT

Repeated measures AN(C)OVAs with AldAR measures over all five measurement timepoints after awakening (0–60 min) revealed a borderline significant group-by-time interaction [*F* (3.94, 228.56) = 2.14, *p* = .077, *η_p_*^2^ = .04, *f* = .19, see Figure 2] that was not independent of confounders (*p *=* *.22). Complementary univariate AN(C)OVAs with AldAR AUCi's as dependent variable showed borderline significantly higher AUCi scores (Figure 2) in the NT group as compared to EHT [*F* (1, 58) = 2.89, *p *=* *.094, *η_p_*^2 ^= .05, *f *=* *.22], again not independent of confounders (*p *=* *.15).

Post-hoc testing (Table 3) revealed that aldosterone levels 15 min after awakening were borderline significantly higher in EHT (*p *=* *.086, *η_p_*^2 ^=^ ^.05) but not when confounders were controlled (*p *=* *.20). Levels at 30, 45, and 60 min after awakening did not differ between both groups (*p*'s ≥ .34). Separate reanalyses in each group further revealed that NT showed an increase after awakening (main effect time: *p *=* *.002, *η_p_*^2 ^= .14), while EHT did not (*p *=* *.97).

#### 3.4.2 Linear associations with MAP

Repeated measures AN(C)OVAs showed that higher MAP borderline significantly related to overall higher repeated AldAR levels [main effect MAP: *F* (1, 58) = 3.63, *p *=* *.062, *η_p_*^2 ^=^ ^.06, *f *=* *.25; with confounders *F* (1, 54) = 2.92, *p *=* *.093, *η_p_*^2 ^=^ ^.05, *f *=* *.23]. There was no interaction effect with repeated levels, nor an association with AUCi (*p*'s ≥ .29) or with aldosterone levels 15–60 min after awakening (*p*'s ≥ .14).

## 4 Discussion

Despite the well-documented diurnal total aldosterone rhythmicity measured from blood samples (25), salivary free diurnal aldosterone levels, particularly the salivary free aldosterone awakening response, received little attention so far in EHT. Here, we investigated for the first time salivary free AldAR in addition to salivary free AldDay levels in EHT as compared to NT controls. We applied a highly standardized procedure with frequent sampling times in the first hour after awakening and five sampling timepoints between 08:30 and 22:00 h in participants' natural environment. Moreover, we controlled for potentially confounding variables such as sleep-related variables (26), as well as for age and BMI.

In terms of *AldDay*, we found evidence for overall higher free aldosterone levels in HT, both in terms of repeated assessment (main effect of group and interaction group-by-time) as well as aggregated total secretion (AUCg). Our post-hoc analysis revealed that differences were most pronounced at the beginning of the day, in particular at 08:30 and 11:00 h. They became less pronounced at 16:00 h and disappeared in the evening hours until 22:00 h. These kinetics were confirmed by significantly steeper diurnal slopes in EHT. Notably, results of both, group comparisons and linear analyses using MAP, overall suggest that the relationship with higher aldosterone levels may exist along a continuum of MAP levels. Our data confirm the diurnal decline shown for plasma total aldosterone in the four studies that compared repeatedly assessed total aldosterone concentrations between HT and NT participants either every two (29), four (30, 31), and six hours (32). However, while we found significant group differences in salivary free aldosterone, these studies report overall similar total aldosterone plasma levels in both groups except for subgroups with elevated aldosterone. Our findings are in line with results from the hitherto only study that analyzed salivary free aldosterone in EHT as compared to NT (38). In that study aldosterone was assessed at erratical times once in the morning and once in the evening on one single day and median aldosterone levels from the 25th–75th percentiles of the two assessment timepoints were computed. The obtained results similarly pointed to higher salivary free aldosterone levels in EHT as compared to NT (38).

With respect to *AldAR*, we found higher free aldosterone levels immediately at awakening in EHT as compared to NT, while levels 15–60 min after awakening did not significantly differ between groups. Differences in terms of repeated AldAR and aggregated AldAR levels were of borderline significance. These findings point to differences between EHT and NT in that EHT seem to start with higher awakening aldosterone levels, however without further increase after awakening. This indicates a tendency towards a flattened AldAR or even a lack of it in EHT while NT showed the regular AldAR in terms of an increase in response to awakening during the first hour after awakening. The results from our group-based analyses were largely confirmed by linear analyses. We found borderline significantly positive linear associations between MAP and individual aldosterone levels at awakening as well as with repeated measurements of AldAR levels.

An integrative explanation for the outlined evidence may be that in the morning hours and during total daytime, EHT seem to show elevated levels of free but not total aldosterone that includes free and bound fractions. Notably, aldosterone binds to binding proteins, including albumin and CBG (33). The previous findings of predominantly similar aldosterone levels in plasma may point to potential differences in plasma binding factors between HT and NT as a mechanism underlying the higher aldosterone daytime levels in HT. However, future studies should clarify these aspects. Moreover, in healthy participants, ACTH increases have been observed in response to awakening (51, 52) and aldosterone release is regulated by ACTH (53). Given this, it is assumable that potential differences in circadian ACTH between EHT and NT may add to the observed higher aldosterone levels in our EHT. However, to the best of our knowledge, ACTH levels in response to awakening have not yet been compared between EHT and NT.

Implications of our study include that altered and in particular higher biologically active free aldosterone levels in terms of AldAR or AldDay may have a corresponding impact on basal daytime BP levels pointing to a potential biological mechanism underlying EHT. Indeed, such reasoning is supported by prospective studies that found higher plasma and serum aldosterone levels to be associated with BP elevation and risk of HT development after four (8, 9) and five years (10). However, cross-sectional studies comparing basal plasma and serum aldosterone levels in NT and EHT report inconsistent results (see Introduction). One reason for these findings might relate to the different methodological approaches e.g., sampling without consideration of daytime and temporal distance to awakening. In future studies, circadian patterns should be taken into account by repeated assessments instead of analysis of single measurements or (urinary) aggregated aldosterone metabolites. Another reason might be that plasma aldosterone consists of bound and non-bound aldosterone whereas salivary aldosterone reflects the non-bound i.e., active fraction of circulating aldosterone. Consequently, our results might indicate higher biologically active aldosterone concentrations in EHT. To address such reasoning, future EHT research is needed that includes parallel assessment of aldosterone from both plasma and saliva to capture not only the bound but also the biologically active free fraction of aldosterone. Moreover, future studies should also assess morning renin levels in addition to plasma and saliva aldosterone given that low renin levels have been associated with the development of HT, the severity of HT, the presence of HT-mediated organ damage, and incidence of cardiovascular events (54–57). Notably, as we did not measure plasma renin and aldosterone, we cannot rule out that plasma aldosterone indeed does not differ from salivary aldosterone. Given this, an alternative explanation for our findings of elevated aldosterone in EHT may relate to potential subclinical primary aldosteronism in an EHT subgroup since recent studies support that primary aldosteronism is not only limited to populations with severe HT but that subclinical primary aldosteronism can also exist in mild HT and NT and thus also in apparently EHT (55, 58–60).

We can only speculate that the magnitude and alterations of salivary free AldAR and AldDay kinetics might also relate to CVD risk. For example, by affecting platelets, coagulation, and fibrinolysis aldosterone levels might contribute to enhanced thrombosis (61) thus increasing the risk for cardiovascular events that commonly occur in the morning hours (62). Clinical implications for EHT diagnosis and management may include to extend the regular HT diagnosis and surveillance by additional assessment of circadian salivary free aldosterone profiles in the context of primary care. For example, primary care physicians may routinely assess salivary aldosterone profiles by instructing HT patients to collect saliva samples at defined timepoints during the day.

*Strengths* of our study comprise the use of a standardized two-day sampling protocol and consideration of potential confounders of aldosterone secretion, including group-matching for age. Moreover, adherence with the study protocol was ensured by the use of MEMS TrackCaps combined with self-recording of sampling times. Also, our study allowed for high ecological validity due to the assessment of participants in their natural environment. This procedure also allowed us to exclude white coat and masked HT. However, our study also has its *limitations*. It is a major limitation of our study that although we measured creatinine, sodium, potassium, and calcium in our EHT, and despite our extensive health questionnaire, we cannot completely rule out all forms of secondary HT in our apparently EHT. Therefore, our diagnosis of EHT may have been compromised to “apparently” EHT. In addition, we did measure aldosterone in saliva only which may impoverish the interpretation of our results. The additional assessment of aldosterone and renin from blood would have allowed for a more extensive RAAS assessment in order to strengthen our interpretation of the observed salivary aldosterone findings in EHT. A more detailed diagnosis would have been needed to identify participants with potential (subclinical) primary aldosteronism as a potential subgroup of apparently EHT. Moreover, the generalizability of our results is limited to NT and apparently EHT but otherwise healthy and medication-free white men. Also, our sample size is relatively small although sufficient, based on our a-priori power calculation. Therefore, large scale studies are needed to test whether our results are generalizable to women and population groups with greater variety in terms of age, socioeconomic status, and ethnicity, as well as clinical populations including those with CVD. A final limitation is that in 20 participants aldosterone data of only one day were available.

In sum, we systematically characterized for the first time the free salivary AldAR and AldDay in EHT as compared to NT. We found evidence for overall increased salivary free aldosterone levels in EHT with increased awakening levels, lack of further increase in response to awakening, and a steeper diurnal decline. Whether elevated salivary free aldosterone levels may point to a biological mechanism underlying EHT and whether subclinical primary aldosteronism plays a potential role in EHT remain to be elucidated. Furthermore, the labeling “essential” indicates the absence of an underlying primary disease and might be reconsidered since at least a sub-group of apparently EHT patients might have an aldosterone-mediated HT. Moreover, the clinical utility of repeated assessments of free aldosterone over the course of the day for EHT diagnosis and prognosis needs to be further investigated.

## Data Availability

The raw data supporting the conclusions of this article will be made available by the authors, without undue reservation.
